# RSV Vaccine with Nanoparticle-Based Poly-Sorbitol Transporter (PST) Adjuvant Improves Respiratory Protection Against RSV Through Inducing Both Systemic and Mucosal Humoral Immunity

**DOI:** 10.3390/vaccines12121354

**Published:** 2024-11-29

**Authors:** Seong-Mook Jung, Soo Ji Kim, Young Chae Park, Eun Sang Seo, Cheol Gyun Kim, Taewoo Kim, Sumin Lee, Eunjin Cho, Jun Chang, Cheol-Heui Yun, Byoung-Shik Shim, In Su Cheon, Young Min Son

**Affiliations:** 1 Department of Systems Biotechnology, Chung-Ang University, Anseong 17456, Republic of Korea; jsun0218@naver.com (S.-M.J.); dudco987@naver.com (Y.C.P.); tjdmstkd123@naver.com (E.S.S.); 2 Laboratory Sciences Division, International Vaccine Institute, Seoul 08826, Republic of Korea; sooji.kim@ivi.int (S.J.K.); taewoo_kim@g.harvard.edu (T.K.); byoungshik.shim@ivi.int (B.-S.S.); 3 Department of Agricultural Biotechnology, and Research Institute of Agriculture and Life Sciences, Seoul National University, Seoul 08826, Republic of Korea; cjfrbs73@snu.ac.kr (C.G.K.); cyun@snu.ac.kr (C.-H.Y.); 4 Graduate School of Pharmaceutical Sciences, Ewha Womans University, Seoul 03760, Republic of Korea; tcell@ewha.ac.kr; 5 Division of Pulmonary and Critical Care Medicine, Department of Medicine, Mayo Clinic College of Medicine and Science, Rochester, MN 55905, USA; auq2aj@virginia.edu

**Keywords:** respiratory syncytial virus (RSV), vaccination, adjuvant, poly-sorbitol transporter (PST), nanovaccine, systemic humoral immune response, mucosal immunity

## Abstract

**Background/Objectives:** Respiratory syncytial virus (RSV) causes symptoms similar to a mild cold for adults, but in case of infants, it causes bronchitis and/or pneumonia, and in some cases, mortality. Mucosal immunity within the respiratory tract includes tissue-resident memory T (T_RM_) cells and tissue-resident memory B (B_RM_) cells, which provides rapid and efficient protection against RSV re-infection. Therefore, vaccine strategies should aim to generate mucosal immune responses. However, the interactions between RSV vaccines and mucosal immune responses within the respiratory tract are poorly understood. We evaluated a mucosal immune system following immunization by RSV vaccine with poly-sorbitol transporter (RSV-PST), a nanoparticle adjuvant. **Methods:** We intranasally immunized the RSV-PST and identified the systemic and mucosal immune responses. Furthermore, we challenged with RSV A2 strain after immunization and investigated the protective effects. **Results:** Consequently, antigen-specific CD8^+^ T_RM_ cells were markedly elevated in the lung parenchyma, yet exhibited impaired cytokine expression. In contrast, humoral immunity, with systemic antibody production from serum, but not in the respiratory tract, was significantly increased by RSV-PST immunization. Interestingly, the production of respiratory mucosal antigen-specific IgG after RSV A2 challenge dramatically increased in the bronchoalveolar lavage fluid (BALF) of the RSV-PST immunized group in the presence of FTY720, and the lung-infected RSV titer was significantly lower in this group. Furthermore, after RSV A2 challenge, CD69^+^ IgG^+^ B_RM_ cells were significantly increased in lung tissues in the RSV-PST group. **Conclusions:** The RSV-PST vaccine has protective effects against RSV infection by promoting both systemic and local humoral immunity rather than cellular immunity.

## 1. Introduction

Respiratory syncytial virus (RSV) is an infectious respiratory virus that targets the lung epithelium, causing symptoms similar to a mild cold [1,2]. Although RSV infection is not a significant concern for adults, it remains a potentially lethal virus for infants, as it can lead to bronchitis and/or pneumonia via lower respiratory tract infection [3,4]. RSV caused 33 million acute lower respiratory tract infections, 3.6 million hospitalizations, and 26,300 fatalities worldwide in 2019 [5]. Only two commercially available RSV vaccines, Arexvy, GSK’s RSVPreF3 vaccine for those aged 60 years and older, and Abrysvo, Pfizer’s RSVpreF vaccine for those who have completed 32–36 gestational weeks or infants aged under 6 months, were approved by the U.S FDA in 2023 [6,7,8]. However, these vaccines that have been approved for use against RSV are only administered via the systemic route. Because the significance of mucosal vaccines, which stimulate local mucosal immunity against particularly respiratory virus infection, has been increased, efficient RSV mucosal vaccines need to be developed.

Respiratory virus infection, including influenza A virus, SARS-CoV-2, and RSV, has been reported to induce the development of tissue-resident memory T (T_RM_) cells and tissue-resident memory B (B_RM_) cells within the respiratory tract, which serve as the first line of defense against pathogen re-infection [9,10,11]. Upon virus re-infection, CD8^+^ T_RM_ cells rapidly produce the IFN-γ and promote cytolytic response to infected cells in situ [12,13]. Furthermore, the B_RM_ cells residing in mucosal sites are quickly reactivated and differentiate to antibody-secreting cells upon exposure to a virus that was already experienced [14,15]. Therefore, the development of T_RM_ and B_RM_ cells in the respiratory tract is one of the crucial objectives when designing mucosal vaccines against respiratory virus infection [16,17,18]. In addition, the intranasal immunization, which mimics the route of virus invasion into the host respiratory system to elicit T_RM_ and B_RM_ cells in the lung mucosal site, is becoming another vaccine strategy to promote mucosal immunity [19,20].

Adjuvants for the RSV vaccine have been developed to efficiently enhance the immunogenicity and host protection against RSV re-infection [21,22,23]. For instance, the intramuscular immunization of RSV-F subunits with the adjuvant system 02 (AS02), the combination of AS03 and Quillaja saponaria fraction 21 (QS-21), induced significantly higher production levels of RSV-F specific antibodies in sera than other adjuvants and provided host protection against the RSV challenge [24,25,26]. In another study, the subcutaneous immunization of a co-delivery system of RSV-F trimers and a TLR-7/8 adjuvant applied with polymer nanoparticles induced the sufficient production of neutralizing antibodies in sera and provided host protection against the RSV challenge [27]. Although those studies of current RSV vaccines with adjuvants have reported the systemic host protection, the development of adjuvants for RSV vaccine-induced mucosal immune responses within the respiratory tract remains poorly understood.

Poly-sorbitol transporter (PST) is a polyethylenimine (PEI)-based polymeric nanocarrier, which is cross-linked with sorbitol diacrylate (SDA) to reduce the toxicity of PEI [28]. As an adjuvant, PST has been reported to promote the cross-presentation within antigen-presenting cells (APCs) through the proton sponge effect of PEI, subsequently eliciting the CD8^+^ T cell responses [29,30]. Furthermore, PST was reported to enhance the antigen-specific antibody responses compared to an administration with antigens alone [28,31,32]. When PST was combined with antigens via electrostatic interaction, the complexes facilitated the stable delivery of antigens and antigen uptake into APCs [28,33,34]. Thus, PST has the potential to be a superior mucosal vaccine adjuvant compared to other adjuvants because of its multiple functions that are favorable for inducing immune responses, with high utilization and safety.

In this context, we hypothesized that intranasal RSV-PST immunization might induce mucosal immune responses and effectively protect the host against RSV re-infection.

## 2. Materials and Methods

### 2.1. Mice

Female BALB/c mice, 6–8 weeks old, were purchased from Orient Bio Inc. (Seongnam, Republic of Korea) and maintained under pathogen-free conditions in animal facility at the International Vaccine Institute (Seoul, Republic of Korea), where they received food and water ad libitum. All protocols used in the animal experiments were approved by the Institutional Animal Care and Use Committees (IACUC) of Chung-Ang University (Seoul, Republic of Korea) (Approval number: A2022068) and International Vaccine Institute (Approval number: PN 2023-017).

### 2.2. Preparation and Characterization of RSV-PST

PST was synthesized with low molecular weight (LMW: 600 Da) PEI and SDA by Michael addition reaction as previously described [28,31]. In brief, SDA and PEI were dissolved in dimethyl sulfoxide (DMSO) at concentrations of 0.836 M and 0.209 M, respectively. The SDA solution was added dropwise to PEI at a feed molar ratio of SDA:PEI = 4:1 upon gentle stirring. The reaction mixture was continuously stirred at 80 °C for 24 h, then dialyzed using a dialysis membrane (Spectra/Pro^®^ membrane, 3500 Da MW cut-off) against distilled water (DW) at 4 °C, lyophilized, and stored at −70 °C until use.

PST complexed with recombinant RSV A2 strain Fusion glycoprotein (RSV F, SinoBiological, Beijing, China) was determined by dynamic light scattering (DLS) and Transmission Electron Microscope (TEM). Briefly, RSV-PST complexes were prepared at various weight ratios (1:1, 1:5, 1:10, 1:20) by incubating the components at room temperature (RT) for 30 min with a final protein concentration of 500 µg/mL for DLS or 40 µg/mL for TEM. For DLS, the particle sizes of the RSV-PST complexes were measured by a DLS-7000 spectrophotometer (Otsuka Electronics, Osaka, Japan). For TEM, a single drop of complexes was placed on the copper grid and stained with 1% uranyl acetate solution for 10 s followed by extensive washing. The grid was dried for 10 min and observed by TEM (JEM-2100PLUS, Carl Jeol Ltd., Kyoto, Japan).

### 2.3. Intranasal Immunization and RSV Challenge

In experiments for immunization assay, BALB/c mice were divided into four groups: (1) control (Ctrl.), (2) RSV-F protein only (RSV-F), (3) RSV-PST, and (4) RSV-F protein with cholera toxin (RSV-CT). The mice were anesthetized with 2.5–3% isoflurane for 4 min using Apparatus Table-Top Anesthesia System for Small Rodents (Harvard Apparatus, Holliston, MA, USA) and immunized intranasally with 10 µg of RSV-F protein alone or RSV-PST including 10 µg of RSV-F for twice on days 0 and 14. As a positive control, 10 µg of RSV-F protein mixed with 1 µg of CT (list labs, Campbell, CA, USA) was immunized. The volume for intranasal immunization in each mouse was 30 µL in PBS.

In experiments for a challenge assay, BALB/c mice were divided into five groups: (1) Ctrl., (2) RSV-PST, (3) RSV-CT, (4) RSV-PST with FTY720, and (5) RSV-CT with FTY720. The mice were anesthetized and immunized as described. On day 34 post first immunization until euthanasia, FTY720 (1 mg/kg; Cayman Chemical, Ann Arbor, MI, USA) was administrated daily by intraperitoneal (i.p.) injection. On day 35 post first immunization, the mice were anesthetized with isoflurane as described and intranasally infected with a high dose of 3 x 10^6^ plaque-forming units (PFU) of RSV A2 (VR-1540, ATCC, Manassas, VA, USA) per mouse, which are required to induce viral replication in mice [35,36,37,38].

### 2.4. Sample Collection

A total of 2 µg of α-CD45-violetFluor™(vF) 500 antibody (clone: 30-F11; Tonbo Biosciences, San Diego, CA, USA) diluted in 300 µL of PBS was administrated via intravenous (i.v.) injection to a tail vein 5 min before mice sacrifice.

For the collection of bronchoalveolar lavage fluid (BALF) and lung, mice were anesthetized with 300 µL of 2.5% avertin ([2,2,2-Tribromoethanol and 2-Methyl-2-butanol]; Sigma-Aldrich, St. Louis, MO, USA) by i.p. injection. The BALF was obtained by three times of lavages with 1 mL of PBS and centrifuged to divide the BALF cells and supernatant. To obtain the single cells of lung tissues, a dissected lung was chopped into small pieces and incubated with collagenase type 2 (183 U/mL; Worthington Biochemical, Lakewood, NJ, USA) in plain Iscove’s Modified Dulbecco’s Medium (IMDM) (Gibco, Grand Island, NY, USA) at 37 °C and 5% CO_2_ for 40 min. Cells were further homogenized via a 70 µm cell strainer (SPL) and washed with plain IMDM. After red blood cell lysis with 1 mL of 1X RBC Lysis Buffer (Invitrogen, Waltham, MA, USA) for 1 min, cells were centrifuged and resuspended in cold plain IMDM.

For challenge experiments, at 4 days post-infection (d.p.i.), blood was obtained from the retro-orbital plexus of anesthetized mice. Next, the left lung was processed as described to obtain single cells for intracellular cytokine profiling with re-stimulation. The right lobes were mashed in 1 mL of serum-free Dulbecco’s modified Eagle’s medium (DMEM) (Gibco) using a 70 µm strainer. The supernatant of mashed lung tissue was used for plaque assay.

### 2.5. Flow Cytometry Analysis

For the analysis of RSV-F specific CD8^+^ T_RM_ cells, the cells from the BALF and lung were stained with PE-labeled H-2K^d^-F_85–93_ (KYKNAVTEL) RSV-F tetramer in FACS buffer for 40 min at RT in dark condition. The RSV-F tetramer was kindly provided by Dr. Jun Chang’s laboratory (Division of Life & Pharmaceutical Sciences, and the Center for Cell Signaling & Drug Discovery Research, Ewha Womans University, Seoul, Republic of Korea). Then, cells were washed with FACS buffer and were stained with CD69-FITC (clone: H1.2F3, Biolegend, San Diego, CA, USA), CXCR6-PE/Cyanine7 (clone: SA051D1, Biolegend), CD103-Brilliant Violet (BV) 421 (clone: 2E7, Biolegend), CD8a-BV605 (clone: 53-6.7, Biolegend), and PD-1-BV711 (clone: 29F.1A12, Biolegend) in FACS buffer for 30 min at 4 °C in dark condition. For eosinophil infiltration assay into BALF, CD11c-FITC (clone: N418, BioLegend), Siglec-F-PE (clone: S17007L, BioLegend), and CD45-APC (clone: 30-F11, BioLegend) were stained in FACS buffer for 30 min at 4 °C in dark condition. For analysis of memory B (B_MEM_) cells, CD4-PerCP/Cyanine5.5 (clone: RM4-5, Biolegend), CD8-PerCP/Cyanine5.5 (clone: 53-6.7, Biolegend), F4/80-PerCP/Cyanine5.5 (clone: BM8, Biolegend), TER-119-PerCP/Cyanine5.5 (clone: TER-119, Biolegend), CD69-FITC (clone: H1.2F3, Biolegend), GL7-PE/Cyanine7 (clone: GL7, Biolegend), CD38-APC (clone: 90, Biolegend), CD45-Alexa Fluor^®^ 700 (clone: 30-F11, Biolegend), CD45R/B220-APC/Cyanine7 (clone: RA3-6B2, Biolegend), IgA-Biotin (clone: RMA-1, Biolegend), Streptavidin-BV421 (Biolegend), IgM-BV510 (clone: RMM-1, Biolegend), IgG-BV605 (clone: Poly4053, Biolegend), and IgD-BV711 (clone: 11-26c.2a, Biolegend) were stained in FACS buffer for 30 min at 4 °C in dark condition. After antibody staining, cells were determined by Attune™ NXT, Acoustic Focusing Cytometer (Invitrogen). Data were analyzed by FlowJo software (version 10.10.0; Tree Star, Ashland, WI, USA).

For intracellular cytokine profiling, the lung cells were re-stimulated with RSV-F protein (2 µg/mL) in complete IMDM (IMDM, 10% FBS, 0.0572 mM 2(β)-mercaptoethanol; Sigma-Aldrich, and Pen Strep Glutamine; Gibco) at 37 °C and 5% CO_2_ for 13 h. Brefeldin A Solution (Biolegend) and Monensin Solution (BioLegend) were added and incubated an additional 5 h. Then, cells were washed with FACS buffer and were stained with surface staining markers including CD4-PerCP/Cyanine5.5 (clone: RM4-5, BioLegend), CD44-APC/Cyanine7 (clone: IM7, BioLegend), CD45-vF500 (clone: 30-F11; Tonbo Biosciences), and CD8a-BV605 (clone: 53-6.7, BioLegend) in FACS buffer for 30 min at 4 °C in dark conditions. For intracellular staining, cells were fixed with Fixation Buffer (BioLegend) for 40 min at RT and permeabilized with Intracellular Staining Perm Wash Buffer (BioLegend) for 40 min at RT. Then, cells were stained with IL-4-PE (clone: 11B11, BioLegend), granzyme B-PerCP/Cyanine5.5 (clone: QA16A02, BioLegend), TNF-α-PE/Cyanine7 (clone: MP6-XT22, Biolegend), IL-17A-APC (clone: TC11-18H10.1, BioLegend), and IFN-γ-BV421 (clone: XMG1.2, BioLegend) in Intracellular Staining Perm Wash Buffer for 1 h at RT in dark condition. Finally, cells were washed and resuspended with Intracellular Staining Perm Wash Buffer. After antibody staining, cells were determined and analyzed as described.

### 2.6. Quantitative RT-PCR

The total RNA of the lung homogenates was extracted using High Pure RNA Tissue Kit (Roche, Basel, Switzerland) according to the manufacturer’s instructions. Random primers (0.3 μg/μL; Invitrogen), dNTP Mix (10 mM; Promega, Madison, WI, USA), DL-Dithiothreitol (DTT) (0.3 M; Promega), and M-MLV Reverse Transcriptase (200 U; Promega) were used to synthesize complementary DNA (cDNA) using Biometra Tone thermocycler (Analytik Jena, Jena, Germany). cDNA was amplified with SYBR^®^ Green Realtime PCR Master Mix (TOYOBO, Osaka, Japan) using QuantStudio™ 1 Real-Time PCR System (Applied Biosystems by Thermo Fisher Scientific, Waltham, MA, USA). The primer sequences of cytokines are listed as follows.

*Hprt*-F: CTCCGCCGGCTTCCTCCTCA, *Hprt*-R: ACCTGGTTCATCATCGCTAATC.

*Il1b*-F: GGTCAAAGGTTTGGAAGCAG, *Il1b*-R: TGTGAAATGCCACCTTTTGA.

*Il2*-F: CCTGAGCAGGATGGAGAATTACA, *Il2*-R: TCCAGAACATGCCGCAGAG.

*Il7*-F: GTGCCACATTAAAGACAAAGAAG, *Il7*-R: GTTCATTATTCGGGCAATTACTATC.

*Ifng*-F: TTCTTCAGCAACAGCAAGGC, *Ifng*-R: CGACTCCTTTTCCGCTTCCT.

*Il4*-F: AGATCATCGGCATTTTGAACG, *Il4*-R: TTTGGCACATCCATCTCCG.

*Il17a*-F: TTTAACTCCCTTGGCGCAAAA, *Il17a*-R: CTTTCCCTCCGCATTGACAC.

### 2.7. Enzyme-Linked Immunosorbent Assay (ELISA)

RSV-F specific antibodies in BALF and serum were measured by ELISA. Nunc™ MaxiSorp™ ELISA Plates (BioLegend) were pre-coated with RSV-F protein (1 µg/mL) in PBS overnight at 4 °C. Plates were washed three times with wash buffer (PBS containing 0.05% of Tween 20; Sigma-Aldrich) and blocked with 200 µL of blocking buffer (PBS containing 3% of bovine serum albumin (BSA); Sigma-Aldrich) for 1 h at RT. Serially diluted BALF supernatant or serum samples in blocking buffer were added to the plates and incubated for 2 h at RT. Plates were washed three times with wash buffer and horseradish peroxidase (HRP)-conjugated goat anti-mouse IgG (1:5000) or IgA (1:1000) (Invitrogen) was added. After incubation for 2 h at RT, plates were washed six times with wash buffer and then 100 µL of tetramethylbenzidine (TMB) Substrate (BioLegend) was added for development. The reaction was stopped by adding 50 µL of Stop Solution (BioLegend). The absorbance at wavelength 450 nm was measured by Spectramax 190 microplate reader (Molecular Device, San Jose, CA, USA).

### 2.8. Plaque Assay

HEp-2 cells, which are highly susceptible to RSV infection, allowing the virus to easily infect and replicate within the cells, were used for viral titration [39]. Monolayers of 90% confluent HEp-2 cells in a 6-well plate were washed with PBS. The HEp-2 cells were inoculated with 10-fold serial dilutions of supernatant from the lung homogenates. After incubation at 37 °C and 5% CO_2_ for 1 h, the supernatant was removed and 0.6% SeaPlaque agarose (Lonza, Basel, Switzerland) with 0.05% neutral red solution (Sigma-Aldrich) was added. After incubation for 4 h, the plaque numbers for each well were counted and normalized to the weight (g) of the lung.

### 2.9. Histology

For hematoxylin and eosin (H&E) staining, the left lobe of the lung was fixed in a 10% of formalin solution (Sigma-Aldrich). Specimen preparation and slide scanning were commissioned to DooYeol Biotech (Seoul, Republic of Korea).

### 2.10. Quantification and Statistical Analysis

All statistical analyses were performed using GraphPad Prism, version 10.2.3 (GraphPad Software) and the results were presented as means ± SEM. Unpaired two-tailed Student’s *t*-test (two group comparisons), ordinary one-way analysis of variance (ANOVA) with Tukey’s multiple comparisons test (multiple group comparisons), and ordinary two-way ANOVA with Tukey’s multiple comparisons test (single pooled variance) were used for data analysis. * *p* < 0.05, ** *p* < 0.01, *** *p* < 0.001 and **** *p* < 0.0001.

## 3. Results

### 3.1. Intranasal RSV-PST Immunization Generates Significantly Higher Lung Memory CD8^+^ T Cells with Tissue Residency than Circulating Memory CD8^+^ T Cells

First, we formulated the RSV-PST nanovaccine to generate mucosal immune responses as a mucosal vaccine candidate. The 20–300 nm size range of nanoparticles is suitable for endocytic antigen uptake by APCs [40,41,42]. Thus, we mixed PST and RSV-F proteins at different weight ratios (1:1, 1:5, 1:10, and 1:20) to find the appropriate weight ratio that would generate nanoparticles with a size of 20–300 nm. At the 1:20 weight ratio, RSV-PST with a size of about 200 nm was synthesized (Supplementary Figure S1A). Furthermore, the polydispersity index (P.I.) of RSV-PST synthesized with weight ratio of 1:20 was less than 0.3, indicating that the RSV-PST was monodisperse [43,44]. In addition, after 30 min in water, RSV-PST complexes formed the stable nanosized particles at weight ratio of 1:20 (Supplementary Figure S1B). These results demonstrated that PST optimally complexed with the RSV-F proteins at weight ratio of 1:20.

**Figure 1:**
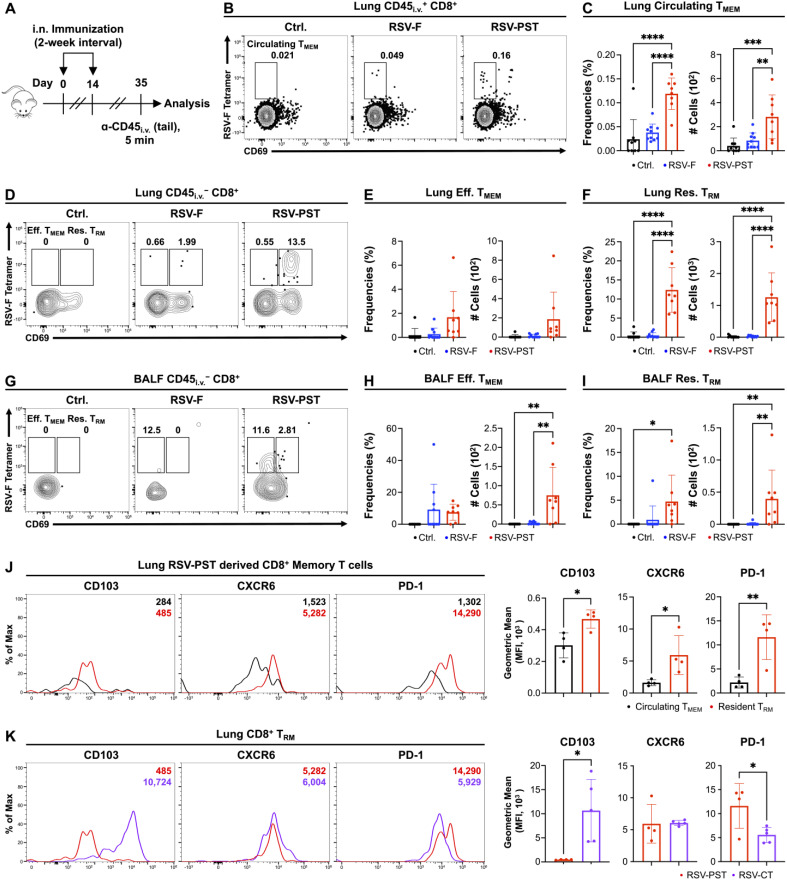
Intranasal RSV-PST immunization generates bona fide RSV-F specific CD8^+^ T_RM_ cells in the respiratory tract. BALB/c mice were intranasally immunized with PBS, RSV-F protein only, RSV-PST, or RSV-CT at 2-week intervals. Mice were i.v. injected with α-CD45 antibody 5 min before sacrifice on day 35 post first immunization. (**A**) Schematic of the experimental design. (**B**) Representative dot plots, (**C**) frequencies, and cell numbers of lung RSV-F-specific circulating T_MEM_ cells (CD45_i.v._^+^ CD8^+^ CD69^−^ RSV-F Tetramer^+^). (**D**) Representative dot plots of lung RSV-F-specific effector T_MEM_ (CD45_i.v._^−^ CD8^+^ CD69^−^ RSV-F Tetramer^+^) or resident T_RM_ cells (CD45_i.v._^−^ CD8^+^ CD69^+^ RSV-F Tetramer^+^). Frequencies and cell numbers of lung RSV-F-specific (**E**) effector T_MEM_ and (**F**) resident T_RM_ cells. (**G**) Representative dot plots of BALF RSV-F-specific effector T_MEM_ or resident T_RM_ cells. Frequencies and cell numbers of BALF RSV-F-specific (**H**) effector T_MEM_ cells and (**I**) resident T_RM_ cells. (**J**) Representative histogram (left) and expression of tissue resident markers (right) in lung RSV-F-specific circulating T_MEM_ or resident T_RM_ cells induced by intranasal RSV-PST immunization. (**K**) Representative histogram (left) and expression of tissue resident markers (right) in lung RSV-F-specific resident T_RM_ cells induced by intranasal RSV-PST or RSV-CT immunization. Data in (**C**,**E**,**F**,**H**,**I**) were pooled from two independent experiments (n = 4–5 per group). *p* values in (**C**,**E**,**F**,**H**,**I**) were analyzed by ordinary one-way ANOVA. Dot plots or histograms in (**B**,**D**,**G**,**J**,**K**) were representative data from two independent experiments (n = 4–5 per group). *p* values in the bar graphs of (**J**,K) were analyzed by unpaired two-tailed Student’s *t*-test. Data are means ± SEM. * *p* < 0.05, ** *p* < 0.01, *** *p* < 0.001 and **** *p* < 0.0001.

Next, we aimed to determine whether the PST adjuvant contributes the generation of RSV-F-specific CD8^+^ T_RM_ cells in the lung parenchyma and airway. To this end, we intranasally primed and boosted the RSV-PST vaccine at 2-week intervals (Figure 1A). On day 35, we administered anti-CD45 antibody (α-CD45) intravenously (i.v.) for 5 min before sacrifice to distinguish tissue-resident cells (CD45_i.v._^−^) and circulating leukocytes (CD45_i.v._^+^) [45,46]. Subsequently, we analyzed the subtypes of CD8^+^ memory T cells in the lung and BALF (Supplementary Figure S2A,B). The cell numbers of total CD45_i.v._^+^ circulating or CD45_i.v._^−^ tissue resident CD8^+^ T cells in the lung were no differences between all groups (Supplementary Figure S2C–E). In addition, the intranasal RSV-PST immunization did not induce the antigen specific CD8^+^ memory T cells in spleen (Supplementary Figure S2F). However, both the frequency and cell numbers of BALF total CD45_i.v._^−^ tissue resident CD8^+^ T cells were significantly increased only in the RSV-PST group (Supplementary Figure S2G,H). Next, to measure the subtypes of RSV-F specific memory CD8^+^ T cells, we utilized CD69, one of the representative tissue-resident marker [47,48], and RSV-F_85–93_ (KYKNAVTEL) tetramer [49,50]. At the results, the significant numbers of RSV-F specific circulating CD8^+^ T_MEM_ cells in the lung were observed only in the RSV-PST group compared to the control or RSV-F groups (Figure 1B,C). Furthermore, RSV-PST immunization led to a dramatic induction of RSV-F specific effector CD8^+^ T_MEM_ cells and RSV-F-specific CD8^+^ T_RM_ cells compared to the control or RSV only group in the lung parenchyma (Figure 1D–F) and BALF (Figure 1G–I). Interestingly, the cell numbers of lung RSV-F-specific CD8^+^ T_RM_ cells were ten times higher than lung circulating CD8^+^ T_MEM_ cells and BALF CD8^+^ T_RM_ cells in the RSV-PST group (Figure 1B–I). Thus, the intranasal immunization of RSV-PST provides significant effects to generate RSV-F-specific CD8^+^ memory T cell subtypes in the lung parenchyma and airway, particularly RSV-F-specific CD8^+^ T_RM_ cells.

Although CD69 serves as a primary marker of tissue residency, CD8^+^ T_RM_ cells express several other tissue-resident markers, including CD103, CXCR6, and PD-1 [51,52,53]. CD103, an αE integrin, interacts with E-cadherin on epithelial cells in mucosal areas, facilitating the residency of CD103^+^ T_RM_ cells generated post-infection or vaccination in mucosal tissues such as the lung, gut, and skin [54,55,56,57]. CXCR6, a chemokine receptor that interacts with CXCL16, plays a role in maintaining T_RM_ cells within the respiratory tract by replenishing the CD8^+^ T_RM_ cells in the airway, whereas PD-1, a T cell inhibitory molecule, reduces the over-activation and maintains the exhausted condition of T_RM_ cells in the mucosal area [58,59]. On that basis, we investigated the tissue-resident phenotypes of RSV-PST-induced CD69^+^ CD8^+^ T_RM_ cells. Lung RSV-PST-induced CD8^+^ T_RM_ cells exhibited significantly higher levels of CD103, CXCR6, and PD-1 expression than circulating CD8^+^ T_MEM_ cells within the lung (Figure 1J). To compare the modality of expression of tissue-resident markers with other type of CD8^+^ T_RM_ cells, we immunized the mice with RSV-F protein mixed with cholera toxin (CT) (RSV-CT) as a positive control, which is a representative and intensive adjuvant for intranasal immunization due to its non-viscosity and high immunogenicity, but is unfavorable to use in practice due to its toxicity in vivo [28,60,61]. The intranasal RSV-CT immunization induced significant numbers of RSV-F-specific CD8^+^ T_RM_ cells in the lung (Supplemental Figure S2I). RSV-PST induced CD8^+^ T_RM_ cells expressed lower levels of CD103 compared to RSV-CT-induced CD8^+^ T_RM_ cells, whereas the expression of CXCR6 was similar in both groups (Figure 1K). Oppositely, PD-1 expression was higher in RSV-PST induced CD8^+^ T_RM_ cells than RSV-CT-induced CD8^+^ T_RM_ cells. This result indicates that each adjuvant PST or CT potentially involved different pathways for induction of lung tissue-resident CD8^+^ T_RM_ cells. Thus, these results suggest that intranasal RSV-PST immunization induces the significant numbers of RSV-F-specific CD8^+^ T_RM_ cells expressing multiple tissue-resident markers in the lung parenchyma.

### 3.2. Deficient Cytokine Productions in CD8^+^ T_RM_ Cells Induced by RSV-PST Immunization

**Figure 2:**
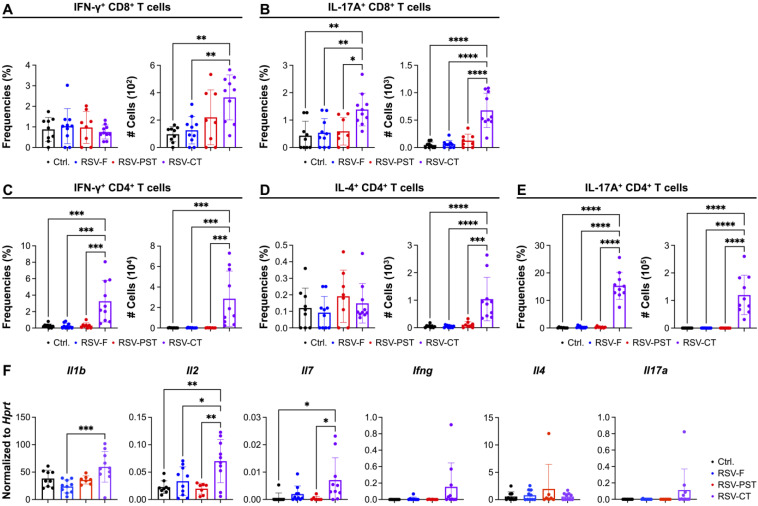
Deficient cytokine production in the lung RSV-F-specific CD8^+^ and CD4^+^ T cells induced by intranasal RSV-PST immunization. After intranasal immunizations with PBS, RSV-F protein only, RSV-PST or RSV-CT, single cells from lung tissue were re-stimulated with RSV-F protein for 18 h and the cytokine production of T cells was analyzed by flow cytometry. Frequencies and cell numbers of lung (**A**) IFN-γ^+^ or (**B**) IL-17A^+^ activated CD8^+^ T cells and lung (**C**) IFN-γ^+^, (**D**) IL-4^+^ or (**E**) IL-17A^+^ activated CD4^+^ T cells. (**F**) The levels of cytokine gene expression in whole lung tissues were measured by qRT-PCR. All data were pooled from two independent experiments (n = 4–5 per group). *p* values were analyzed by ordinary one-way ANOVA. Data are means ± SEM. * *p* < 0.05, ** *p* < 0.01, *** *p* < 0.001 and **** *p* < 0.0001.

To identify whether the RSV-PST induces CD8^+^ T_RM_ cells provide general memory function with cytokine productions, we investigated the production of functional cytokines after re-stimulation with RSV-F protein. Surprisingly, the RSV-PST immunization showed no effects on production of any functional cytokines including IFN-γ and IL-17A in the CD8^+^ T cells, whereas the RSV-CT group induced sufficient amounts of cytokines in the CD8^+^ T cells (Figure 2A,B and Supplementary Figure S3A). Furthermore, the production of IFN-γ, IL-4, and IL-17A cytokines in CD4^+^ T cells was only observed in the RSV-CT group but not the RSV-PST group (Figure 2C–E and Supplementary Figure S3B).

We next sought to identify cytokines that were associated with memory T cell generation and maintenance in the lung environment after immunization with RSV-PST. The whole lung mRNA expression levels of *Il1b* involved in immunological activation [62], and *Il2* and *Il7*, which are known to be required for the formation and homeostasis of T_RM_ cells [63,64,65,66], were only significantly increased in the RSV-CT group, whereas the RSV-PST immunization showed no differences compared to the control group (Figure 2F). These data are consistent with the failure to generate functional cytokines in T_RM_ cells upon RSV-F protein re-stimulation after RSV-PST immunization. The mRNA expression levels of T cell cytokines including *Ifng*, *Il4*, and *Il17a* did not increase relative to the control, because T_RM_ cells have few cytokine responses in the steady state without any stimulation [67,68]. Taken together, these results indicate that RSV-F-specific T_RM_ cells generated by RSV-PST immunization exhibit a deficiency in the production of functional cytokines that are essential for the development of memory responses.

### 3.3. Intranasal RSV-PST Immunization Induces Significant Systemic Humoral Immunity

**Figure 3:**
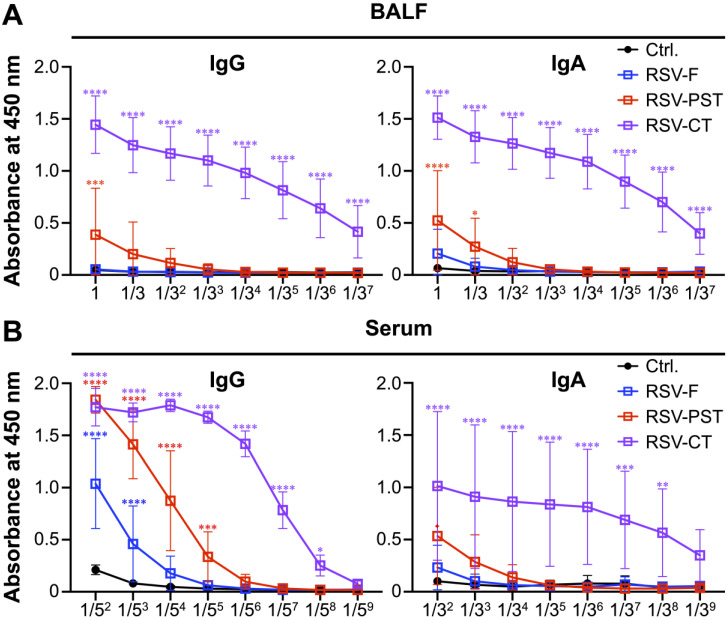
Intranasal RSV-PST immunization induces the production of systemic RSV-F-specific IgG. BALF and serum were harvested on day 35 from the intranasally immunized mice with PBS, RSV-F protein only, RSV-PST, or RSV-CT at 2-week intervals. The production levels of RSV-F-specific IgG and IgA in serial diluted (**A**) BALF and (**B**) serum were measured by ELISA. All data were pooled from two independent experiments (n = 4–5 per group). *p* values were analyzed by ordinary two-way ANOVA with Tukey’s multiple comparisons test (single pooled variance). Symbol * indicates a comparison to control group. Data are means ± SEM. * *p* < 0.05, ** *p* < 0.01, *** *p* < 0.001, and **** *p* < 0.0001.

Next, to identify B cell-mediated immune responses, the production levels of RSV-F-specific IgG and IgA in BALF and serum were examined. We initially assessed the humoral immune responses in the lung mucosal area using the BALF. RSV-F-specific IgG and IgA production was less in BALF of the RSV-PST group than those in the RSV-CT group, similar to the control and RSV-F groups (Figure 3A and Supplementary Figure S4A,B,E,F). In contrast, when comparing the amount of RSV-F-specific antibodies in serum, which provides protection against systemic infection, RSV-PST induced the significantly higher levels of production of RSV-F-specific IgG compared to the those in the control and RSV-F groups, but not IgA (Figure 3B and Supplementary Figure S4C,D,G,H). Consequently, the intranasal immunization of RSV-PST potentially contributes to host protection by which enhancing systemic humoral immunity.

### 3.4. The Immunization of RSV-PST Provides Sufficient Host Protection Against RSV Challenge Through the Enhancement of Mucosal Antibody Production

**Figure 4:**
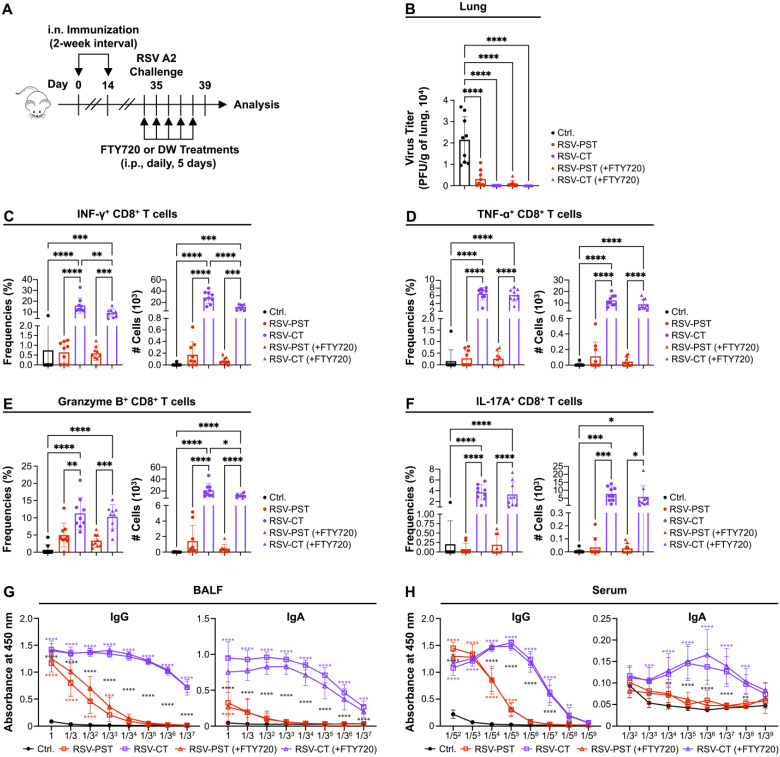
Intranasal RSV-PST immunization provides mucosal protection against RSV A2 challenge by inducing systemic and local antigen-specific IgG production. BALB/c mice were immunized with intranasal administration of PBS, RSV-PST, or RSV-CT at 2-week intervals and challenged with RSV A2 strain (starting at day 35 post first immunization, intranasal route) in the presence or absence of FTY720 treatment (starting at day 34 post first immunization, i.p. route). The lung tissues were harvested and analyzed at 4 d.p.i. (**A**) Schematic of the experimental design. (**B**) Virus titers of RSV A2 strain within the lung tissues were measured by plaque assay. Frequencies and cell numbers of lung (**C**) IFN-γ^+^, (**D**) TNF-α^+^, (**E**) granzyme B^+^ (GrB), or (**F**) IL-17A^+^-activated CD8^+^ T cells that were re-stimulated with RSV-F protein for 18 h. The production levels of RSV-F-specific IgG and IgA in serial diluted (**G**) BALF and (**H**) serum were measured by ELISA. All data were pooled from two independent experiments (n = 4–5 per group). *p* values in (**B**–**F**) were calculated by ordinary one-way ANOVA. *p* values in (**G**,**H**) were analyzed by ordinary two-way ANOVA with Tukey’s multiple comparisons test (single pooled variance). A red or purple symbol * indicates a comparison to the control group. A black symbol * indicate a comparison between the RSV-PST and RSV-CT groups. Data are means ± SEM. * *p* < 0.05, ** *p* < 0.01, *** *p* < 0.001 and **** *p* < 0.0001.

We hypothesized that RSV-F-specific IgG induced by RSV-PST immunization may contribute to host protection against RSV re-infection. Additionally, we wondered whether RSV-F-specific IgG is produced in the respiratory mucosal area when blocking the migration of lymphocytes from the blood to lung, in response to RSV re-infection. To this end, the mice were immunized with RSV-PST or RSV-CT and then challenged with the RSV A2 strain on day 35 post the first immunization in the presence or absence of FTY720 treatment (Figure 4A). FTY720 was used to minimize the effect of peripheral lymphocytes by preventing the migration of lymphocytes from the blood to lung tissues [46,69,70]. RSV A2 challenge induced no changes in body weight in any group over 4 days (Supplementary Figure S5A), because RSV A2 was known to have non-pathogenic effects in mouse models [71,72]. Furthermore, since the RSV infection induces the type 2 immune response and infiltration of eosinophils, we measured the infiltration of eosinophil into the airway to confirm the lung pathology caused by RSV infection [73,74,75]. However, airway infiltration of eosinophils did not occur regardless of immunization (Supplementary Figure S5B,C). In addition to this, histological analysis showed that RSV A2 infection did not cause local inflammation in the mice system regardless of immunization (Supplementary Figure S5D). These all results demonstrate that the RSV A2 does not cause pathology in mice system.

Next, to estimate the protective responses against RSV A2 challenge in the RSV-PST or RSV-CT immunized groups, the virus titer of RSV within the lung tissue was determined by plaque assay at 4 d.p.i. The results demonstrated a dramatical reduction in infectious virus titer in the RSV-PST groups compared to the non-immunized control group (Figure 4B and Supplementary Figure S5E). Additionally, the viral clearance ability was comparable to that observed in the RSV-CT group, which served as a positive control. Moreover, the presence or absence of FTY720 treatment did not affect the significant reduction of virus titer in the lung. Therefore, this result indicates that local mucosal memory responses are sufficient to provide host protection by RSV-PST immunization against the RSV A2 challenge.

Next, we determined whether lung RSV-F-specific CD8^+^ memory T cells are re-activated in response to the RSV A2 challenge. To this end, a re-stimulation experiment was conducted using RSV-F protein. Consistent with previous cytokine production results from the RSV-PST-immunized model (Figure 2), the RSV-PST immunization showed no functional roles to induce cytokines including IFN-γ, TNF-α, granzyme B (GrB), IL-4, and IL-17A in the CD8^+^ and CD4^+^ T cells unlike the RSV-CT group (Figure 4C–F and Supplementary Figure S6A–E). However, surprisingly, the production of RSV-F specific IgG in the BALF was dramatically increased in the RSV-PST group compared to the control, whereas IgA was still comparable to the control group, similar to the result in Figure 3A (Figure 4G and Supplementary Figure S7A,B,E,F). Furthermore, the increase in IgG production levels in the BALF from RSV-PST immunization was similar in the FTY720 present or absent groups (Figure 4G and Supplementary Figure S7A,E). Additionally, the lung CD69^+^ IgG^+^ B_RM_ cells were dramatically increased in the RSV-PST-immunized group compared to control group (Supplementary Figure S8A,B). However, the frequencies of lung and spleen CD69^−^ IgG^+^ B_MEM_ cells as well as other IgA^+^ B_RM_ or B_MEM_ were comparable to the control group (Supplementary Figure S8C–G). These results indicate that the memory responses of the local humoral immunity, especially IgG^+^ B_RM_ cells, but not cell-mediated immunity, re-activated by the RSV A2 challenge are sufficient to produce the lung-local antigen-specific IgG, which may provide host protection. The serum RSV-F specific IgG production, but not IgA, was increased by RSV-PST immunization in the presence or absence of FTY720 treatment (Figure 4H and Supplementary Figure S7C,D,G,H), and this suggests that serum systemic IgG contributes to host protection against RSV A2 challenge.

In addition, we investigated whether RSV-PST immunization provides similar protection against RSV A2 re-infection in both genders of mice model. In RSV-PST groups of both genders, not only the infectious virus titers were significantly reduced (Supplementary Figure S9A), but also the RSV-F specific IgG in BALF and serum was highly produced compared to the control groups (Supplementary Figure S9B–E). These data suggest that RSV-PST immunization provides protection against the RSV A2 challenge regardless of gender.

Together, our results suggest that intranasal RSV-PST immunization induces to produce systemic antigen-specific antibodies and contributes to host mucosal protection against RSV infection. Furthermore, intranasal RSV-PST immunization may induce the mucosal memory response of humoral immunity, which potentially contributes to host mucosal protection against the RSV A2 challenge.

## 4. Discussion

A recent study revealed that acute RSV infection greatly induced lung T_RM_ cells for a long period and these local T_RM_ cells provided superior protective ability against viral challenge [76]. These results support the importance of local mucosal immunity including T_RM_ cells and B_RM_ cells for further RSV vaccine strategies.

Thus, we identified the marked induction of T_RM_ cells in the lung parenchymal after RSV-PST vaccine immunization. Our results demonstrated that the expression level of CD103 was lower in RSV-PST-induced CD8^+^ T_RM_ cells compared to RSV-CT-induced CD8^+^ T_RM_ cells (Figure 1K). Previous studies reported that the generation of lung CD103^+^ CD8^+^ T_RM_ cells was supported by CD4^+^ T cell-derived IFN-γ after influenza viral infection [54,55]. However, the RSV-PST vaccine had no effect on the induction of IFN-γ^+^ CD4^+^ T cells after antigen re-stimulation whereas the RSV-CT immunization induced IFN-γ-producing CD4^+^ T cells in the lung parenchyma (Figure 2C). Therefore, the difference of IFN-γ^+^ CD4^+^ T cell generation between the RSV-PST and RSV-CT immunization may induce the opposite expression of CD103 in CD8^+^ T_RM_ cells. Next, the RSV-PST induced a higher expression of PD-1 than the RSV-CT on CD8^+^ T_RM_ cells (Figure 1K). PD-1 is an immunoinhibitory receptor that suppresses T cell activation induced by TCR stimulation of T cells [77]. During chronic viral infection, the increasing expression levels of PD-1 on T cells induces functional exhaustion [78,79]. Although PD-1 is generally observed in T_RM_ cells post-viral infection, excessive PD-1 expression is more likely to induce exhausted T cells. Taken together, the increasing PD-1 expression may affect the functional deficiency in intranasal RSV-PST immunization-induced pulmonary RSV-F-specific CD8^+^ T_RM_ cells (Figure 2A,B).

Interestingly, intranasal RSV-PST immunization failed to induce the functional activation of RSV-F-specific CD8^+^ T_RM_ cells; however, it contributed to producing the systemic and local RSV-F-specific IgG and provided mucosal protection against the RSV A2 challenge (Figure 4B,G–H). The RSV-PST nanovaccine enhanced antigen delivery efficiency [28,32] and production of RSV-F specific IgG through humoral immune responses without local CD4^+^ T cell help (Supplementary Figure S6B–E). Several studies reported that such CD4^+^ helper T cell-independent pathogen-specific antibodies could provide sufficient protection. For example, mice lacking CD40 or CD4^+^ T cells resulted in detectable levels of influenza-specific IgG after influenza virus infection, providing a similar level of protection against influenza virus re-infection comparable to that of WT mice [80]. In addition, follicular helper T (T_FH_) cell-lacking *Bcl6*^fl/fl^*Cd4*^Cre^ mice generated durable antigen-specific antibodies, which were exhibited high affinity and neutralizing capacity against various SARS-CoV-2 variants after immunization with the SARS-CoV-2 vaccine [81]. Our results demonstrate that intranasal RSV-PST immunization induced the production of systemic RSV-F-specific IgG in serum (Figure 3B and Figure 4H). Furthermore, RSV-PST immunization in a presence of FTY720 treatment produced the mucosal RSV-F-specific IgG against RSV A2 challenge and local IgG^+^ B_RM_ cells were significantly increased after RSV-PST immunization and RSV A2 challenge, suggesting that the mucosal RSV-F-specific IgG might be produced by local B cells presented within the pulmonary mucosal site (Figure 4G and Supplementary Figure S8B). In this context, the humoral immune responses without the assistance of CD4^+^ helper T cells potentially provide adequate protection against pulmonary RSV infection.

The PST adjuvants facilitate antigen delivery to the respiratory tract through a simple formulation of antigen–PST complexes. Although we did not directly compare the capability to other nanoparticle-based adjuvants, our data indicate that the intranasal RSV-PST immunization could induce the humoral immune response and provide the host protection against respiratory RSV infection similar to CT adjuvant, which are the intensive adjuvant for respiratory vaccines (Figure 4B). Furthermore, the PST adjuvant was confirmed to be safer than CT by measuring infiltration of eosinophils (Supplementary Figure S5B,C). Additionally, the PST adjuvants could improve the responses of cytotoxic T lymphocytes via cross-presentation of dendritic cells (DCs) and induce antibody responses via interaction between CD4^+^ T cells and DCs [29,30].

In our study, there are some limitations. Although we demonstrated that the mucosal CD69^+^ IgG^+^ B_RM_ cells and RSV-F-specific IgG antibodies provided sufficient protection against RSV infection, we have not directly identified the generation of RSV-F-specific B cells or B_RM_ cells in pulmonary mucosal sites via intranasal RSV-PST immunization due to the technical limitation of generating RSV-F epitope-specific tetrameric complexes. In a future study, we are going to focus more on identifying the characteristics and cellular mechanisms of pulmonary local B cell responses, including germinal center B cells, plasma cells, and B_RM_ cells post RSV-PST immunization and RSV-A2 challenge. In addition, the comparison of vaccine effectiveness between systemic and intranasal routes needs to be discussed. We should consider these issues in our future study.

## 5. Conclusions

In summary, RSV-PST immunization induced the production of the systemic and mucosal RSV-F-specific IgG antibodies and significantly increased the local IgG^+^ B_RM_ cells after RSV A2 challenge, whereas the production of functional cytokines was not induced in lung T_RM_ cells. Furthermore, those RSV-F-specific IgG and local IgG^+^ B_RM_ cells were involved in host protection against RSV infection. In conclusion, our results suggest that the RSV-PST nanovaccine provides sufficient host mucosal protection against RSV infection by promoting systemic and respiratory humoral immune responses rather than local cell-mediated immunity.

## Data Availability

The data presented in this study can be available on request.

## Supplementary Materials

The following supporting information can be downloaded at: https://www.mdpi.com/article/10.3390/vaccines12121354/s1, Supplementary Figure S1. Characterization of PST with RSV-F protein.; Supplementary Figure S2. Intranasal RSV-PST immunization induces the recruitment of CD8+ T cells into the respiratory track.; Supplementary Figure S3. The lung RSV-F specific T cells induced by intranasal RSV-PST immunization lack the adequate function of cytokine expression.; Supplementary Figure S4. The production of RSV-F specific antibodies induced by intranasal RSV-PST immunization.; Supplementary Figure S5. Pathology of RSV A2 infection in mice system.; Supplementary Figure S6. Deficient cytokine expression of lung RSV-F specific T cells after RSV A2 challenge.; Supplementary Figure S7. The production of RSV-F specific antibodies induced by intranasal RSV-PST immunization after RSV A2 challenge.; Supplementary Figure S8. Intranasal RSV-PST immunization induces the lung CD69^+^ IgG^+^ B_RM_ cells in the respiratory tract after RSV A2 challenge.; Supplementary Figure S9. Intranasal RSV-PST immunization provides protection against RSV A2 challenge regardless of GENDER.
