# Supplementary figures and images for: RSV Vaccine with Nanoparticle-Based Poly-Sorbitol Transporter (PST) Adjuvant Improves Respiratory Protection Against RSV Through Inducing Both Systemic and Mucosal Humoral Immunity
Source: Vaccines (Basel). 2024 Nov 29;12(12):1354. doi: 10.3390/vaccines12121354 (PMC11680183; doi:10.3390/vaccines12121354)

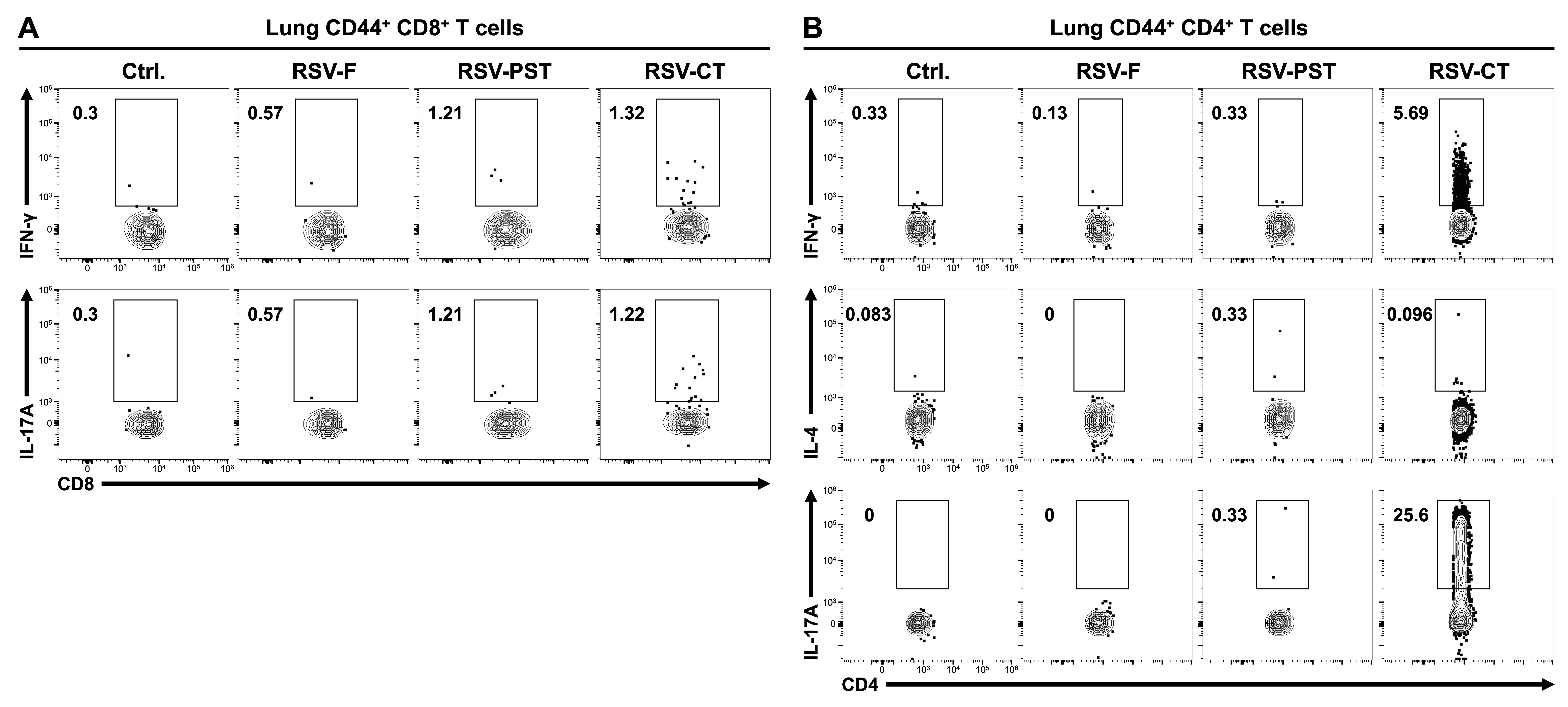

Supplement: Supplementary file 1 [file vaccines-12-01354-s001.zip › Supplement Figure S3.tiff]
